# Binding of Nanoparticles Harboring Recombinant Large Surface Protein of Hepatitis B Virus to Scavenger Receptor Class B Type 1

**DOI:** 10.3390/v13071334

**Published:** 2021-07-10

**Authors:** Shuji Hinuma, Kazuyo Fujita, Shun’ichi Kuroda

**Affiliations:** 1 The Institute of Scientific and Industrial Research, Osaka University, Mihogaoka 8-1, Ibaraki 567-0047, Osaka, Japan; 2 Faculty of Human Life Science, Senri Kinran University, Fujisirodai 5-25-1, Suita 565-0873, Osaka, Japan; k-fujita@cs.kinran.ac.jp

**Keywords:** hepatitis B virus (HBV), surface large protein (L protein), bio-nanocapsule (BNC), scavenger receptor class B type 1 (SR-B1), HEK293T cells, sodium taurocholate co-transporting polypeptide (NTCP), heparan sulfate proteoglycan (HSPG), phosphatidylcholine (PC)

## Abstract

(1) Background: As nanoparticles containing the hepatitis B virus (HBV) large (L) surface protein produced in yeast are expected to be useful as a carrier for targeting hepatocytes, they are also referred to as bio-nanocapsules (BNCs). However, a definitive cell membrane receptor for BNC binding has not yet been identified. (2) Methods: By utilizing fluorescence-labeled BNCs, we examined BNC binding to the scavenger receptor class B type 1 (SR-B1) expressed in HEK293T cells. (3) Results: Analyses employing SR-B1 siRNA and expression of SR-B1 fused with a green fluorescent protein (SR-B1-GFP) indicated that BNCs bind to SR-B1. As mutagenesis induced in the SR-B1 extracellular domain abrogates or attenuates BNC binding and endocytosis via SR-B1 in HEK293T cells, it was suggested that the ligand-binding site of SR-B1 is similar or close among high-density lipoprotein (HDL), silica, liposomes, and BNCs. On the other hand, L protein was suggested to attenuate an interaction between phospholipids and SR-B1. (4) Conclusions: SR-B1 can function as a receptor for binding and endocytosis of BNCs in HEK293T cells. Being expressed various types of cells, it is suggested that functions as a receptor for BNCs not only in HEK293T cells but also in other types of cells.

## 1. Introduction

In the HBV genome, three genes overlapped in a single open reading frame (ORF) encode surface proteins (HBs): large (L protein: pre-S1 + pre-S2 + S regions), middle (M protein: pre-S2 + S regions), and small (S protein: S region) [1]. S protein occupies more than 90% of the hepatitis B surface antigen (HBsAg) whereas L protein is 5–10 times more abundant in HBV virions and HBs filaments than in the HBs spheres [2].

Hollow phospholipid nanoparticles (PNPs) coated with L protein can be produced using yeast (*Saccharomyces cerevisiae*) introduced with a plasmid to express L protein. They have been expected to be useful as a carrier in drug delivery systems (DDS) to convey targeted bioactive substances to hepatocytes in vitro and in vivo. Therefore, they are also referred to as BNCs [3,4]. To use BNCs in vivo, escape mutant-type BNCs (emBNCs) have been developed to reduce the immunogenicity of L protein by the substitution of two amino acid residues (i.e., Q129R and G145R) in the S region. They are expected to be safer carriers than wild-type BNCs for in vivo use [5]. Although BNCs were once expected as a model mimicking HBsAg or HBV particles, the usefulness of BNCs as an HBV model is thought to be limited [6].

Recent studies have revealed that sodium taurocholate co-transporting polypeptide (NTCP), which is encoded by the *SLC10A1* gene, is indispensable as a receptor for HBV to achieve HBV infection into host cells [7]. Although HBV cannot infect a hepatic cell line, HepG2 cells, because these cells do not express sufficient NTCP, can be susceptible to HBV infection by introducing an NTCP expression plasmid [8]. On the other hand, glypican 5 (GPC5), a kind of heparan sulfate proteoglycan (HSPG), is reported as a low-affinity receptor for HBV and plays an important role in the early process of HBV infection together with NTCP [9]. Although the L protein plays an essential role in the recognition of hepatocytes by HBV in the early process of HBV infection, BNCs cannot bind to NTCP because the N-terminal myristoylation of pre-S1 is indispensable for the binding ability of L protein to NTCP [7,9,10]. On the other hand, BNCs are taken up by HepG2 cells with or without expressing NTCP in a pathway independent of NTCP [11]. As heparin can suppress the NTCP-independent endocytosis of BNCs by HepG2 cells, HSPG such as GPC5 is presumed to be involved in this process [11]. However, as a definitive receptor responsible for BNC binding on the cell surface has not been identified yet, there is still ambiguity about precise mechanisms responsible for the receptor-dependent binding and endocytosis of BNCs. 

We have previously demonstrated that PNPs such as liposomes and exosomes are incorporated into various types of mammalian cells including hepatic cells and that they can induce the formation of lipid droplets (LDs) in the cytoplasm [12]. Additionally, we have revealed that liposomes consisting of phosphatidylethanolamine (PE) and phosphatidylcholine (PC), which are major phospholipids in mammalian cell membranes, are efficiently endocytosed via SR-B1 (encoded by *SCARB1* gene) expressed in HEK293T cells [13]. Furthermore, we have recently proven that PC-containing liposomes bind to SR-B1 and that phosphatidic acid (PA) can modulate this [14]. SR-B1 is known as the primary receptor for HDL and can bind not only HDL but also other ligands [15]. Considering the results of our previous studies, it is likely that SR-B1 plays a regulatory role in the binding and endocytosis of various PNPs. Additionally, PC and PE are representative phospholipids in recombinant HBsAg produced in yeast [16,17,18]. Therefore, we presumed that BNCs could bind to SR-B1. In this study, we demonstrate that BNCs can bind to SR-B1 expressed in HEK293T cells.

## 2. Materials and Methods

### 2.1. Preparation of BNCs

BNCs (i.e., we used emBNCs in this study) were prepared as reported previously [6]. Briefly, an expression plasmid, which was constructed by introducing two mutations (Q129R and G145R) in wild-type BNC expression plasmid (pGLDLIIP39-RcT), was transfected into *S. cerevisiae* AH22R^−^. BNCs, produced thereby in yeast, were purified homogeneously by the method, including heat treatment and sulfated Cellulofine column chromatography as reported previously [19]. The amount of protein contained in the BNCs was determined by BCA assay using bovine serum albumin as a standard. Particle sizes, zeta-potentials, and polydispersity indices (PDIs) of BNCs were measured using the Zetasizer Nano ZS (Malvern).

### 2.2. Preparation of Liposomes

Liposomes consisting of 1,2-dioleoyl-*sn*-phosphatidylcholine (DOPC) were prepared as described previously [12,13,14]. Their particle sizes (nm), zeta potentials (mV), and PDIs (n = 3) were 107.4 ± 8.9, −8.7 ± 1.4, and less than 0.3, respectively.

### 2.3. Cell Culture

We maintained HEK293T cells (Riken RBC, Tokyo, Japan), which is a cell line derived from Human Embryonic Kidney 293 (HEK293) cells transfected with SV40 large T antigen, in a culture dish (Thermo Fisher Scientific, Waltham, MA, USA) in 5% CO_2_ at 37 °C using a culture medium: RPMI1640 medium (Gibco, Amarillo, TX, USA) supplemented with antibiotics (penicillin and streptomycin; Nacalai Tesque, Kyoto, Japan) and 10% heat-inactivated fetal bovine serum (FBS; Sigma-Aldrich, St. Louis, MO, USA).

### 2.4. Fluorescent Labeling of BNCs and Liposomes 

(i) In flow cytometric analyses to detect BNC binding and endocytosis, BNCs were labeled with CellVue Claret (CV) (Molecular Targeting Technologies Inc., West Chester, PA, USA) according to the method described previously [14]. Briefly, 1 mM of CV in ethanol was added to the suspension (about 100 µL of PBS) of BNCs or liposomes (50 µL of CV per 1-mg BNCs) and the suspension was quickly mixed. The resultant mixtures were left to sit at 22 °C for more than 30 min. (ii) In laser scanning microscopy (LSM) to detect BNC binding, we performed fluorescent labeling of BNCs using 500 µL of CV per 1 mg of BNCs because the detection sensitivity of CV-labeled BNC binding using LSM was lower than that using flow cytometry in the present assays. CV labeling was done extemporaneously. After the labeling procedure, more than 99% of CV was estimated to be incorporated into BNCs based on analysis using ultracentrifugation as described previously [13,14].

### 2.5. LSM

(i) Trypsinized HEK293T cells (4 × 10^5^) were incubated with CV-labeled BNCs in PBS (100 µL) in a microtube on ice for 1 h and they were washed twice with cold PBS. Cells were suspended in a small volume of PBS and then an aliquot of the suspension on a cover glass was immediately subjected to LSM (FV-1000, Olympus). (ii) To examine colocalization of CV-labeled BNCs and SR-B1-GFP in BNC binding, HEK293T, SR-B1-HEK, and GFP-HEK cells were precultured for 24 h in the RPMI1640 medium (1 × 10^5^/mL, 300 µL/well) in an 8-well chambered cover glass (Thermo Fisher Scientific) that was coated with poly-L-lysine. After cells were gently washed twice with cold PBS, they were incubated with CV-labeled BNCs at 4 °C for 30 min. Cells were washed twice with cold PBS and they were subjected to LSM immediately.

### 2.6. BNC-Binding Assays Using Flow Cytometry

HEK293T cells were harvested by treatment with a trypsin-EDTA solution (Nacalai Tesque) diluted 4-fold at 22 °C for 1–2 min. Then, they were washed twice with PBS at 4 °C by centrifugation. Fluorescence-labeled BNC or liposomal suspension (100 µL) was admixed to a cell pellet (4 × 10^5^) in a 1.5-mL tube. After an admixture in a tube was incubated on ice for 1 h, cells were washed twice with cold PBS. Then, fluorescence intensity (FI) of 1 × 10^4^ cells was analyzed using a flow cytometer (FACSCant™ II, BD Biosciences). We assessed the binding of BNCs to HEK293T cells from a geometric mean of FI using software (FlowJo™ 7.6.5, BD Biosciences). Their binding to HEK293T cells was calculated by the following formula: (FI of cells treated with fluorescence-labeled BNCs or liposomes) − (FI of untreated cells). Data were expressed as means ± standard errors (vertical bars that were frequently invisible because too small) in triplicate assays. To obtain two-parameter dot plot data, 5 × 10^4^ cells were subjected to analysis.

### 2.7. Competitive BNC-Binding Assays

To examine the inhibitory effect of unlabeled BNCs on the binding of CV-labeled BNCs to HEK293T cells, trypsinized cells (4 × 10^5^) were incubated with unlabeled BNCs in 50 µL of PBS on ice for 1 h and then CV-labeled BNCs in PBS (50 µL each) were admixed to the cell suspension. After the admixture was incubated on ice for 1 h, cells were washed twice with cold PBS by centrifugation and then they (1 × 10^4^) were subjected to flow cytometry. Data are shown as relative values when CV-BNC binding in the absence of unlabeled BNCs was defined as 100%. 

### 2.8. RNA Sequencing (RNA-Seq)

We performed RNA-seq as described previously [13]. Data were indicated as the normalized values of FPKM (Fragments Per Kilobase of transcript per Million mapped reads).

### 2.9. SiRNA Treatment

Treatment of HEK293T cells with siRNA was performed as described previously [13]. Control and SR-B1 siRNAs (Silencer^®^ Select siRNA; negative control #1 and ID: s2650, respectively) were purchased from Thermo Fisher Scientific. To examine the effect of siRNAs on the binding of BNCs, we precultured HEK293T cells (4 × 10^4^/mL) in a 500 µL well in a 24-well plate for 24 h. Transfection of siRNA into HEK293T cells was performed using PolyMag Neo^TM^ (OZ Biosciences, San Diego, CA, USA). A mixture of siRNA (1 pmol) and 0.5 µL of PolyMag Neo™ in Opti-MEM^®^ (50 µL; Gibco) was added to each well and the plate was magnetized for 30 min. Cells treated with siRNA were cultured for an additional 48 h. After cells were washed twice with cold PBS, they were subjected to BNC-binding assays. 

### 2.10. Expression of SR-B1-GFP and Its Mutants in HEK293T Cells

Stable cell lines of HEK293T cells expressing SR-B1-GFP fusion protein (SR-B1-GFP-HEK) and GFP (GFP-HEK) were established by transfecting a pCMV3-C-GFP Spark and control plasmid lacking ORF of SR-B1 [13,14]. Transient expression of SR-B1-GFP, GFP, and SR-B1-GFP mutants (i.e., S112F, T175A, K151A, K156A, M441A/L448A/L455A, and C-deletion) in HEK293T cells was performed using PolyMag Neo^TM^ as described previously [14]. Briefly, A mixture of DNA (0.6–0.8 µg) and 0.6–0.8 µL of PolyMag Neo^TM^ in Opti-MEM^®^ (100 µL) was added to each well of cells cultured in a 24-well microplate, and magnetic treatment was done for 20 min at room temperature. Transfected cells were used for BNC binding assays 24 h after transfection.

### 2.11. BNC Uptake Assays

The uptake of BNCs was performed as described previously [13,14]. Briefly, 6 h after plasmids were transfected into HEK293T cells which were cultured in a 24-well plate, CV-labeled BNCs (final concentration of 5 µg/mL) were added to cells (final 1 mL/well) and then were cultured for 18 h. After cells were harvested by trypsinization, they were subjected to flow cytometry. BNC uptake was calculated from the FI of CV-BNCs incorporated into cells: FI of untreated cells was defined as 1.0 and the uptake of BNCs was expressed as a relative FI (fold) of BNC-treated cells. 

## 3. Results

### 3.1. Binding of BNCs to HEK293T Cells

**Figure 1:**
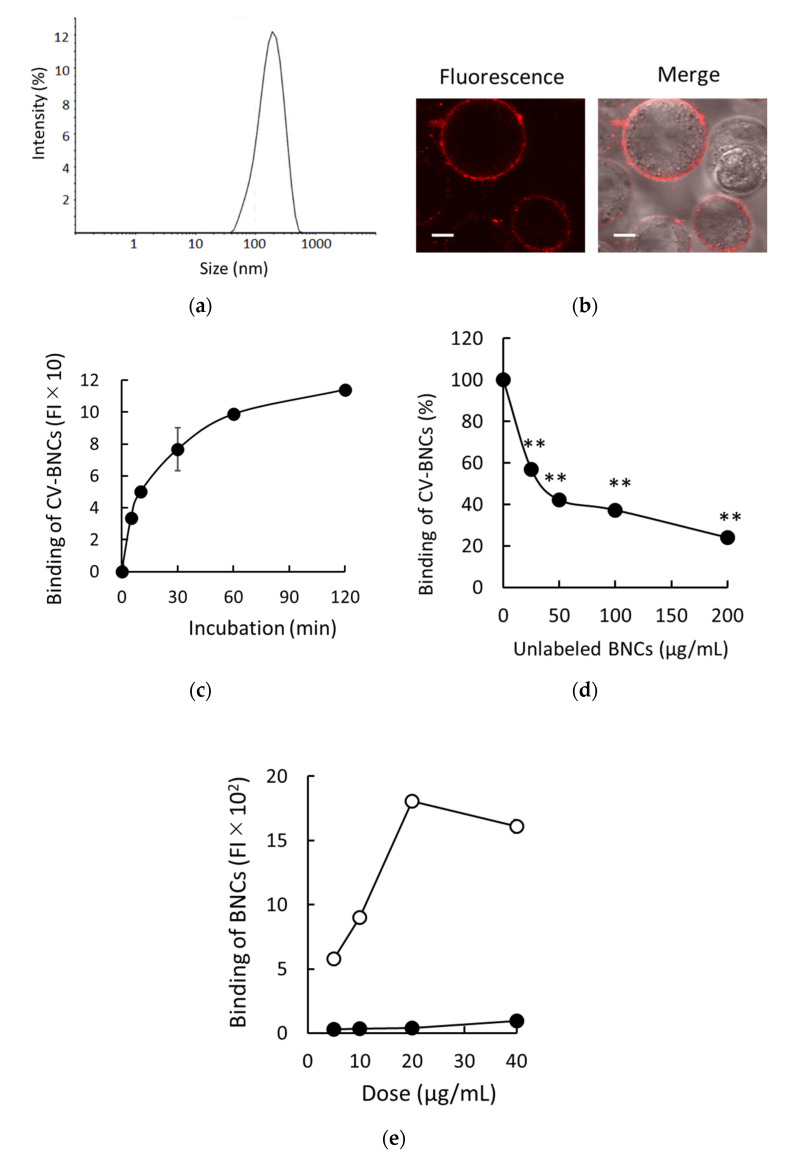
Binding of BNCs to HEK293T cells. (**a**) Size distribution of BNCs. (**b**) Binding of CV-labeled BNCs to HEK293T cell membranes. Trypsinized cells were incubated in the presence of CV-BNCs (100 µg/mL) on ice for 30 min. BNCs bound to cells were detected under LSM. Fluorescence: CV fluorescence is indicated in red; Merge: images of fluorescence and bright field are overlaid. Bars indicate 5 µm. (**c**) Time-dependent binding of BNCs to cells. Cells were incubated with 25 µg/mL of CV-BNCs on ice for 5–120 min. Then, they were subjected to flow cytometry. (**d**) Inhibition of CV-BNC binding to cells by unlabeled BNCs. Cells were incubated with CV-BNCs (25 µg/mL) and unlabeled BNCs at the indicated doses on ice for 1 h and then they were subjected to flow cytometry. Data are shown as a percentage of CV-BNC binding in the presence of unlabeled BNCs to that in the absence of competitors (100%). Statistical analysis was performed using Student’s *t*-test between CV-BNC binding with and without competitors; ** indicates *p* < 0.01. (**e**) Binding of BNCs and DOPC liposomes to HEK293T cells. Indicated doses of BNCs (●) or DOPC liposomes (○) were added to HEK293T cells and their bindings were analyzed using flow cytometry.

We previously developed a method to assess liposomal binding to HEK293T cells using fluorescence-labeled liposomes [14]. Therefore, we applied it to detect the binding of BNCs to HEK293T cells. The representative size distribution of BNCs used in this study is shown in Figure 1a. Sizes (nm), zeta-potential (mV), and PDI of BNCs (n = 3) were 156.9 ± 1.4, −7.8 ± 0.5, and less than 0.2, respectively. As shown in Figure 1b, after incubation of HEK293T cells with CV-BNCs on ice, the patchy and ring pattern of CV-BNCs bound to HEK293T cell surface was detected under LSM. The binding pattern of BNCs on HEK293T cell surfaces was consistent with that of liposomes as reported previously [14]. We subsequently examined the time course of BNC binding. As shown in Figure 1c, BNCs gradually bound to HEK293T cells in a time-dependent manner and almost reached a plateau between 60 and 120 min. Additionally, we tested the competitive inhibition of CV-labeled BNC binding to HEK293T cells by unlabeled BNCs. As shown in Figure 1d, the binding of CV-labeled BNCs to HEK293T cells was inhibited by 1-, 2-, 4-, and 8-fold amounts of unlabeled BNCs in a dose-dependent manner. Although we employed CV-labeled BNCs to assess BNC binding, these results indicate that CV-labeled BNCs bind to HEK293T cells independent of CV, that is, binding of BNCs themselves to HEK293T cells was detected in our assays. The major components of BNCs are L proteins and lipids in which phospholipids are predominant. In recombinant S protein particles derived from yeast, protein-to-lipid ratios are reportedly about 60–70:30–40 [16,17,18]. We have reported that DOPC liposomes efficiently bind to HEK293T cells via SR-B1 [14]. To assess the contribution of phospholipids in BNC binding to HEK293T cells, we compared the binding of BNCs and DOPC liposomes to HEK293T cells at the same doses (weight). As shown in Figure 1e, the binding of DOPC liposomes was more than 10 times greater than that of BNCs. Because BNCs possess the structure of PNPs coated with L proteins, these results strongly suggested that L proteins on the surface of BNCs attenuate the interaction between phospholipids and HEK293T cell membranes.

### 3.2. Binding of BNCs to HEK293T Cells via SR-B1

**Figure 2:**
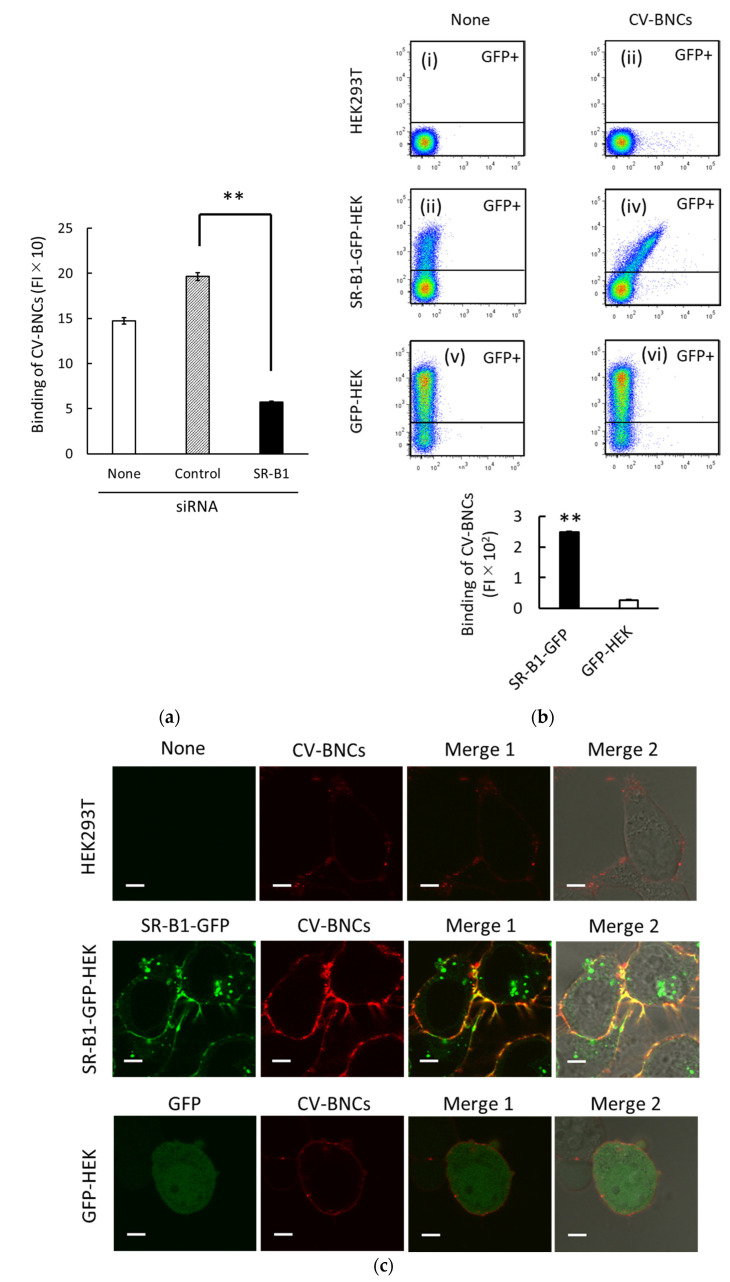
Binding of CV-labeled BNCs to SR-B1. (**a**) Suppression of CV-BNC binding to HEK293T cells by SR-B1 siRNA. After cells were treated with control or SR-B1 siRNA for 48 h, trypsinized cells were incubated with CV-BNCs (25 µg/mL) on ice for 1 h. Binding of CV-BNCs to untreated (open bar), control siRNA-treated (hatched bar), SR-B1 siRNA (closed bar) was measured using flow cytometry. Statistical analysis was performed using Student’s *t*-test; ** indicates *p* < 0.01. (**b**) Binding of CV-BNCs to HEK293T, SR-B1-GFP-HEK, and GFP-HEK cells. In two-parameter dot plots (i–vi), vertical and horizontal axes indicate FI of GFP and CV-BNCs, respectively. In each dot plot, the upper gated area represents the GFP+ subset. The bar graph shows the BNC binding of the GFP+ subset in SR-B1-GFP-HEK (closed bar) and GFP-HEK cells (open bar). Statistical analysis was done in the same manner as (a). (**c**) Co-localization of SR-B1-GFP and CV-BNCs at the membrane of SR-B1-GFP-HEK cells. Binding of CV-BNCs in HEK293T (upper line), SR-B1-GFP-HEK (middle line), and GFP-HEK cells (bottom line) were observed under LSM. The fluorescence of GFP and CV is shown in red and green, respectively; Merge 1: GFP and CV fluorescence images are overlaid; Merge 2: GFP and CV fluorescence and bright filed images are overlaid. Bars indicate 5 µm.

In previous studies, we found that SR-B1 plays an important regulatory role in at least partial binding and endocytosis of liposomes containing various phospholipids (i.e., PC, PE, PS, and PA) in HEK293T cells [13,14]. Since BNCs are mainly composed of phospholipids and L protein, we presumed that SR-B1 would be involved in BNC binding to HEK293T cells too. In HEK293T cells, SR-B1 (*SCARB1*) mRNA was abundantly expressed, i.e., 50.3 FPKM, whereas NTCP (*SLC10A1*) and GPC5 mRNA expressions were <0.1 and 0.9 FPKM, respectively. As shown in Figure 2a, SR-B1 siRNA treatment suppressed BNC binding to HEK293T cells. It should be noted that we have previously confirmed that SR-B1 mRNA and protein expression levels were reduced to around 20% by the SR-B1 siRNA treatment reproducibly under the same experimental conditions. We subsequently examined the binding of BNCs to HEK293T expressing SR-B1-GFP fusion protein (i.e., SR-B1-GFP-HEK cells). Characteristics of this cell line were reported previously [13,14]. As shown in a two-parameter dot plot (iv) in Figure 2b the obvious increase in BNC binding to SR-B1 in proportion to that of GFP expression was detected in the GFP-positive (GFP+) subset of SR-B1-GFP-HEK cells. As the analyses of BNC binding to GFP+ subsets of SR-B1-GFP-HEK and GFP cells are shown in the bar graph of Figure 2b, an evident increase in BNC binding was detected in the GFP+ subset of SR-B1-GFP-HEK cells. As to the bar graph of Figure 2b, the difference of BNC binding between SR-B1-GFP-HEK and GFP-HEK cells appears to be amplified because (i) BNC binding data were obtained from GFP+ subsets (around 20% of the whole cell population) and (ii) SR-B1-GFP in SR-B1-GFP-HEK cells were distributed both at the membrane and cytoplasm (Figure 2c). We examined the localization of CV-BNCs in SR-B1-GFP-HEK, GFP-HEK, and HEK293T cells (Figure 2c). In SR-B1-GFP-HEK cells, the co-localization of SR-B1-GFP and CV-BNCs was observed in the cell membrane whereas cytoplasmic SR-B1-GFP did not co-localize with CV-BNCs. In GFP-HEK cells, co-localization of GFP and CV-BNCs was not detected. Taken together, these data obtained using siRNA and SR-B1-GFP indicated that BNCs can bind to SR-B1.

### 3.3. Binding and Endocytosis of BNCs to HEK293T Cells via SR-B1-GFP Mutants

**Figure 3:**
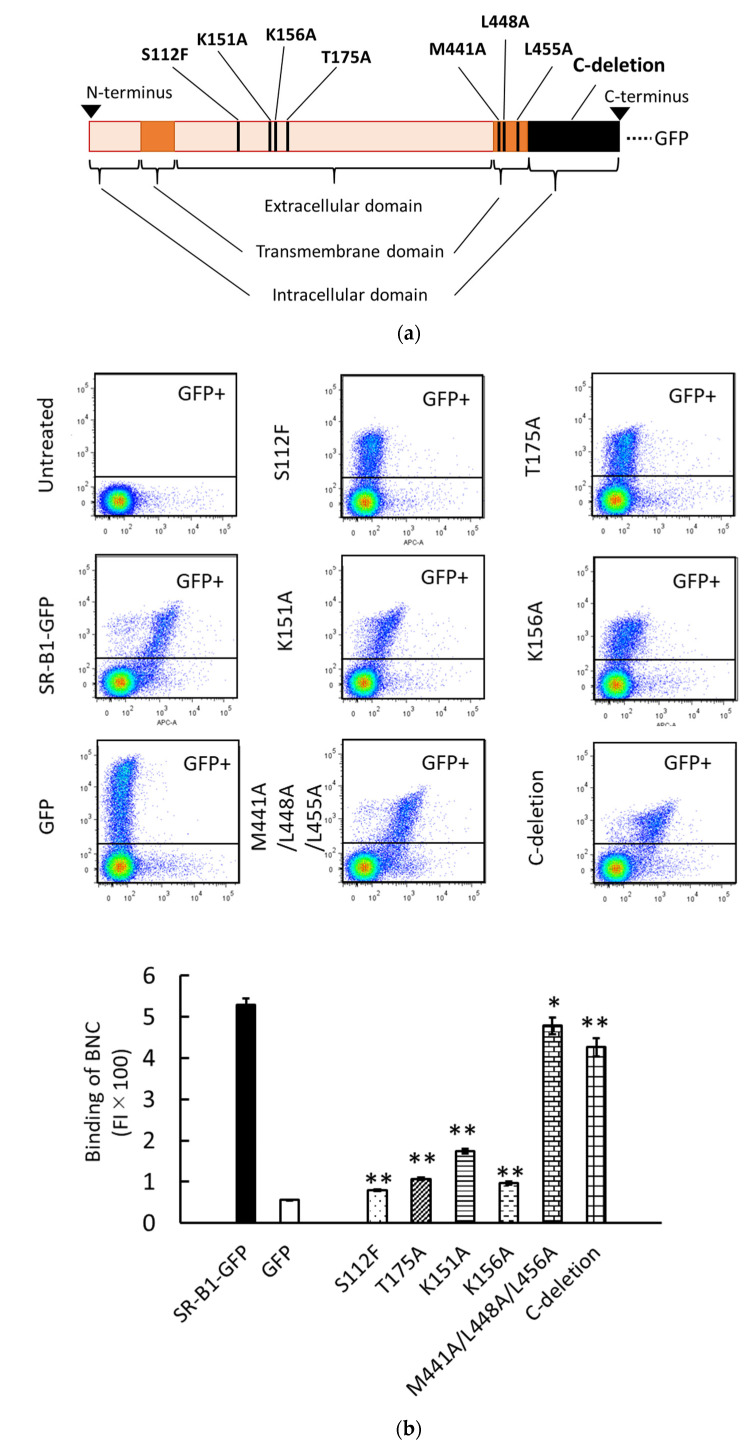

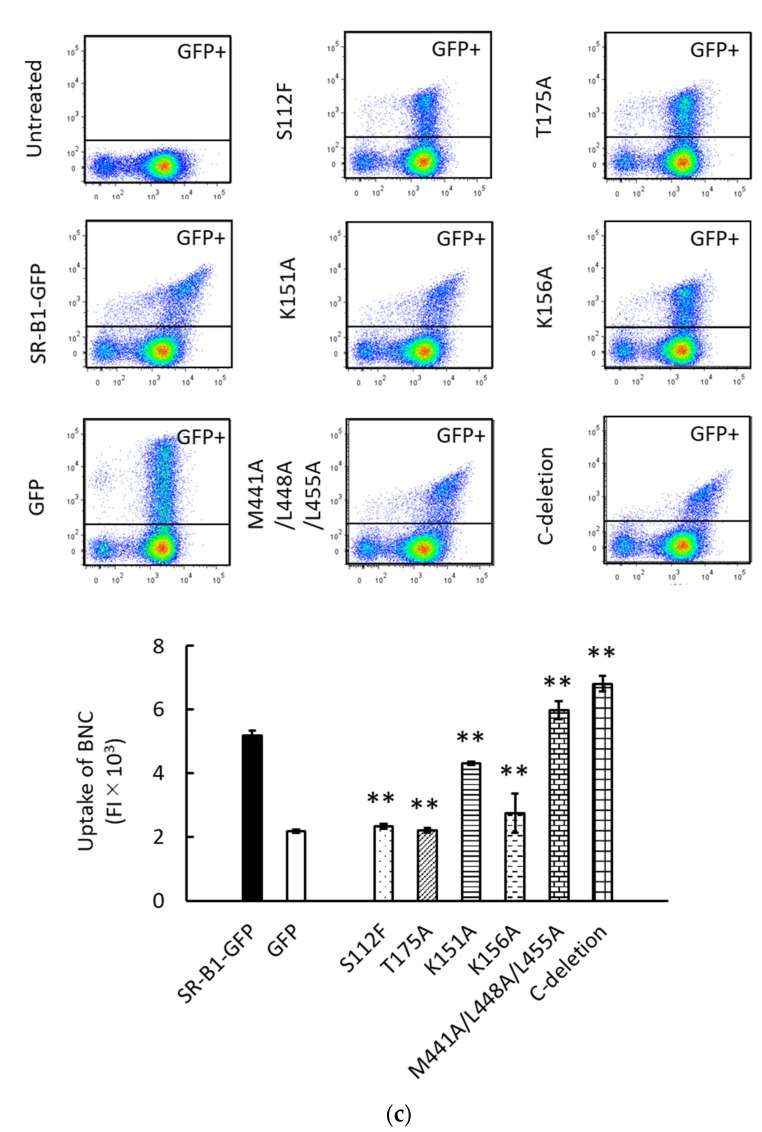
The interaction of BNCs and mutated SR-B1-GFP expressed in HEK293T cells. (**a**) Schematic illustration of mutagenesis induced in SR-B1-GFP. Mutated sites in SR-B1 (462 a.a.) are indicated as vertical black stripes. GFP is fused to the C-terminus of SR-B1. (**b**) Binding of CV-BNCs to HEK293T cells expressing mutated SR-B1-GFP. BNC binding assays using flow cytometry were conducted 24 h after plasmids to express SR-B1-GFP and its mutants were transfected into HEK293T cells. The vertical and horizontal axes of two-parameter dot plots indicate FI of GFP and CV, respectively. The bar graph shows the binding of CV-BNCs to the GFP+ subset (i.e., the upper gated area of dot plots) of plasmid-transfected cells. In triplicate assays, statistical analysis was done between cells expressing the wild type and mutated SR-B1-GFP. (**c**) Uptake of BNCs by HEK293T cells expressing mutated SR-B1-GFP. Cells were cultured for 18 h in the presence of CV-BNCs (5 µg/mL) 6 h after they were transfected with plasmids. Trypsinized cells were then subjected to flow cytometry. Data are shown in the same manner as (**b**). In bar graphs, data were expressed as means ± standard errors (vertical bars) in triplicate assays. Statistical analysis was performed using Student’s *t*-test between HEK293T cells transfected with plasmids to express authentic SR-B1-GFP and mutants; * and ** indicate *p* < 0.05 and *p* < 0.01, respectively.

We have previously analyzed the liposomal binding site of SR-B1 using HEK293T cells transiently expressing SR-B1-GFP mutants [14]. This approach was used to analyze the BNC binding site of SR-B1. The schematic illustration in Figure 3a shows the mutations of SR-B1-GFP used in this study. S112F, T175A, K151A, and K156A are mutations in the extracellular domain of SR-B1. S112F and T175A have been originally discovered as mutations causing loss of HDL-binding activity in SR-B1 [20]. We have demonstrated that both S112F and T175A abolish the binding of liposomes containing PC to SR-B1. K151A and K156A have originally been reported to reduce the binding of silica to SR-B1 [21]. We have reported that K151A and K156A mutations also decrease PC-liposomal binding to SR-B1. M441A/L448A/L455A mutation in the transmembrane domain and the deletion of the C-terminal intracellular domain (i.e., C-deletion) have been expected to alter multimerization and signal transduction of SR-B1, respectively [22,23]. A previous study has shown that these mutations do not profoundly suppress PC-liposomal binding to SR-B1. Western blot analyses regarding the transient expression of SR-B1-GFP and its mutants have been reported previously [14]. As shown in Figure 3b, the four mutations in the extracellular domain drastically reduced the binding of BNCs to SR-B1, although the suppressive effect of K151K is lower than those of the other mutations. These results suggest that the binding site of SR-B1 for BNCs is common or close to those for HDL, silica, and PC-containing liposomes. On the other hand, the effects of mutations in the transmembrane domain and C-terminal intracellular domain were marginal and undetectable, respectively. These results are mostly consistent with those obtained using PC-containing liposomes. The results of the present study strongly suggest that the binding site of SR-B1 is similar or close among BNCs, liposomes, HDL, and silica. Additionally, we examined the effects of these mutations on SR-B1-dependent endocytosis of BNCs by HEK293T cells. As shown in Figure 3c, the mostly consistent effects of the mutations were detected between SR-B1-dependent binding and endocytosis. These results indicated that the interaction of BNCs and SR-B1 can induce the endocytosis of BNCs in HEK293T cells. Although in the BNC uptake assays, we did not distinguish liposomal binding to the cell membranes and uptake into cells, the quantity of membrane-bound BNCs was estimated to be 1% in whole liposomes associated with cells [14].

## 4. Discussion

In this study, we demonstrated that BNCs bind to HEK293T cells via SR-B1, based on the results of experiments using SR-B1 siRNA and the expression of SR-B1-GFP. We have previously revealed that liposomes comprising various phospholipids including PC, PE, and PA bind to SR-B1, and are then endocytosed in HEK293T cells. Therefore, we inferred that BNCs could bind to SR-B1 because the major constituents of BNCs are phospholipids and L protein. As expected, we found that BNCs can bind to SR-B1 in HEK293T cells. However, the results of the present study strongly suggested that L protein attenuates interaction between the phospholipids of BNCs and HEK293T cells. Therefore, it was presumed that L proteins act to reduce at least the interaction between phospholipids in BNCs and SR-B1. It is consistent with the result of our preliminary investigation that pre-S1 peptide did not show apparent binding to SR-B1 (data not shown). Further study will be necessary to reveal which parts of BNCs including the S domain are crucial for BNC binding to SR-B1. So far, BNCs have been demonstrated to be potent carriers to target hepatocytes and the liver [4,5,6,7]. As hepatocytes and sinusoidal endothelial cells in the liver abundantly express SR-B1, the specific targeting activity of BNCs for hepatocytes and the liver could be explained at least in part by the binding of BNCs to SR-B1 [24]. Our preliminary study indicated that HepG2 cells with or without NTCP express SR-B1 more abundantly than HEK293T cells (data not shown). Therefore, we expect that SR-B1 would function as a receptor for BNCs in hepatic cells as well as HEK293T cells. On the other hand, a previous study has revealed that there is a heparin-binding domain (30–42) in the pre-S1 region. This domain has been suggested to play a pivotal role in the specific recognition of hepatic HSPG by BNCs [25]. Considering the results of the previous and present studies together, multiple factors including HSPG and SR-B1 would regulate the specific targeting ability of BNCs for hepatocytes and the liver. 

NTCP is an indispensable receptor for HBV to infect hepatocytes [8,9]. Although the N-terminal myristoylation of L protein is required for L protein to bind NTCP, BNC-derived L protein is not myristoylated. Therefore, BNCs do not have binding ability to NTCP [8,10]. Nevertheless, BNCs as well as myristoylated pre-S1 peptide (2–48), Mry47, can bind a hepatic cell line, HepG2 cells, with or without expressing NTCP [11]. Therefore, it is presumed that there is a receptor(s), which can bind not only HBV or Myr47 but also BNCs, independent of NTCP. However, the following point should be noted regarding Myr47: a very recent study indicated that Myr47 can inhibit the uptake of PNPs into HepG2 cells in a non-specific manner [26], which suggests that the inhibition of BNC uptake into HepG2 cells by Myr47 does not necessarily mean the existence of a pre-S1-dependent BNC endocytic mechanism in HepG2 cells. This presumption dose not contradict the results of the present study that L protein would reduce the interaction between phospholipids of BNCs and SR-B1.

HSPG has been a potential candidate for a BNC receptor because BNC uptake into HepG2 cells can be blocked by heparin [11]. GPC5 is reported as a low-affinity receptor for HBV and it plays an important role in the early process of HBV infection together with NTCP [9]. Therefore, GPC5 has been assumed to be a representative HSPG that can bind BNCs in hepatic cells. However, the expression of GPC5 mRNA expression was substantially lower than that of SR-B1 mRNA in HEK293T cells. Therefore, it was unlikely that GPR5 acts as a major receptor for BNC binding in HEK293T cells. On the other hand, it has been reported that in hepatitis C virus infection, multiple receptor molecules, including SR-B1, low-density lipoprotein receptor, CD81, claudin-1, occluding, epidermal growth factor receptor, and EphA2 are involved in its process. [27]. Therefore, there is a possibility that not only NTCP but also other multiple receptors including SR-B1 participate in the binding and entry of BNCs or HBV in hepatocytes, even though SR-B1 would not play a critical role in HBV infection. Future studies are expected to clarify this point.

## Data Availability

All data generated and analyzed during this study are included in this article.
